# Four-Year Training Course for Police Officers (CFOP) and Fitness Outcomes of Police Academy Cadets: A Cohort Study from 2004 to 2020

**DOI:** 10.3390/healthcare11212901

**Published:** 2023-11-03

**Authors:** Luís Miguel Massuça, Luís Monteiro, Gabriel Coutinho, Vanessa Santos

**Affiliations:** 1 Higher Institute of Police Sciences and Internal Security, 1300-663 Lisbon, Portugal; 2 ICPOL, Higher Institute of Police Sciences and Internal Security, 1300-663 Lisbon, Portugal; 3 First Responder Research Laboratory, University of Kentucky, Lexington, KY 40506, USA; 4 CIDEFES, Lusófona University, 1749-024 Lisbon, Portugal; 5 CIFI2D, Faculty of Sport, University of Porto, 4200-450 Porto, Portugal; 6 Exercise and Health Laboratory, CIPER, Faculty of Human Kinetics, University of Lisbon, 1495-751 Cruz Quebrada, Portugal; 7 KinesioLab, Research Unit in Human Movement Analysis, Instituto Piaget, 2805-059 Almada, Portugal

**Keywords:** aerobic capacity, age, gender, normative data, police academy, speed, strength

## Abstract

This study examines the effect of gender, age, and a 4-year training course for police officers (CFOP) on the physical fitness attributes of Portuguese police academy cadets. This longitudinal cohort study considered 686 police cadets (female, n = 131; male, n = 555 male), corresponding to 2578 fitness assessments (female, n = 509; male, n = 2069). The database of police cadets’ physical fitness evaluations (from 2004/2005 to 2019/2020) comprises body size, speed, agility, strength, flexibility, and aerobic capacity first assessment (T0) and evaluations at the end of the first four years of the CFOP (T1, T2, T3, T4). Results showed that (i) female cadets are younger (*p* < 0.05), shorter, lighter, less fast, less agile, less strong, and perform worse in aerobic capacity assessments than male cadets (all, *p* < 0.001) but perform better in the flexibility assessment (*p* < 0.001); (ii) female cadets > 29 years are significantly heavier, slower, jump less, perform fewer sit-ups, and perform less on the Cooper test (but they have more handgrip strength), and male cadets > 29 years are significantly heavier, slower, jump less, perform fewer sit-ups, and have less flexibility and aerobic capacity (still, they have superior back and lumbar strength and handgrip strength); and (iii) from T0 to T4 (Δ), female cadets are significantly faster (60 m, −0.32 s; slalom, −0.78 s), jump further (+4 cm), have more abdominal strength endurance (+2.6 repetitions) and more back and lumbar strength (+89.8 kg), and male cadets are significantly heavier (+3.27 kg), faster (60 m, −0.23 s; 30 m, −0.15 s; slalom, −0.91 s), jump further (+8 cm), complete more repetitions in the sit-ups (+4.9 repetitions) and in pull-ups (+2.5 repetitions) and have more back and lumbar strength (+92.1 kg) and handgrip strength (+8.6 kg) but a lower aerobic capacity (Cooper test, −74.8 m; *V*O_2_max, −1.3 mL/kg/min) when compared to T0. The study’s findings lead to widely accepted conclusions within the discipline. Nevertheless, this work provides valuable insights into the impact of various factors on the physical fitness of Portuguese police academy cadets, i.e.: (i) it is an essential study with practical implications for recruitment, training, and the ongoing development of Portuguese police academy cadets and police officers; and (ii) these results can also assist in tailoring training programs to different age groups and genders, which is crucial in police training.

## 1. Introduction

The performance of the police function has a physical range of actions, from undemanding situations, such as administrative services, to highly demanding occurrences, such as physical confrontations [1,2], i.e., it can range from sedentary to extremely physically demanding [2,3,4,5]. In this regard, physical fitness can be an essential component of being well-prepared to perform infrequent but critical tasks as a police officer [6,7], and the consistent monitoring of physical fitness should be one of the fundamental pillars for their mission to be successful.

Usually, the importance of physical fitness in police institutions is reflected in recruitment (selection process), training courses (formative years), and performance of professional duties. After the recruitment, police academies prepare cadets to deal with the occupational impacts of the profession, i.e., during the formative period, police academy cadets must develop numerous skills and attributes that will enable them to meet their future profession’s physical and mental challenges [8,9] and to create long-term physical habits, to provide them with physical benefits throughout their careers [10].

Improving the traditional health-related components of physical fitness (i.e., body composition, cardiorespiratory fitness, muscular strength, endurance, and flexibility) is essential to enhancing policing skills and quality of life [11,12]. However, it is essential to highlight the following: (i) male police officers perform significantly better than female police officers on all measures of physical fitness [1,13,14,15,16]; (ii) the significant association between increasing age and decline in physical fitness is known [4,13,14,17,18]; and (iii) that there is a decline in fitness with advancing age—it is noteworthy that strength decreases by 7% to 8% per decade after age 40, and by 25 to 27% at age 70, and aerobic capacity (absolute *V*O_2_max) decreases ~10%/decade after age 30 (however, in athletes who continue to train the decrease is reduced by half, i.e., 5%) [16].

Due to the benefits in health (physical and psychological), profession (success in) and the importance of enforcing exercise habits early in the formative training courses [2], it seems imperative to monitor the physical development of police academy cadets to ensure that physical fitness is being trained correctly.

However, the physical fitness assessment periods are associated with the duration of each police academy’s formative training or the training curriculum being implemented [2,17,19,20,21,22]. Accordingly, it is important to highlight those studies that (i) conduct training programs over a multi-week period and tend to assess the effect of training in a similar manner (before, during, and after the training program) (e.g., [23]), or (ii) for more extended periods, tend to conduct assessments at the beginning and end of each academic semester or year [24]. The monitoring allows for more specific and individualised interventions, corresponding to each academy’s and its cadets’ needs [25].

Physical fitness evaluations can be distributed into three categories, i.e.: (i) distinct physical tests (giving rise to the well-known battery of tests) [18,19,20]; (ii) circuit/job-task simulations [21]; or (iii) hybrid or mixed, being a junction of a simulation and one or more physical fitness tests to assess a specific component of physical fitness [26,27,28].

A recent review of the literature (see [29,30]) showed that a variety of physical fitness tests exist to assess and predict police officer performance, and the most applied fitness assessments were (i) push-ups, sit-ups, vertical jump, and handgrip tests for muscle strength; (ii) 12-min Cooper, 1.5-mile run, 2.4-mile run, and 20 m shuttle run for aerobic capacity; and (iii) sit-and-reach for flexibility. According to Massuça et al. [29], more and more tests are being used to assess various fitness attributes, such as muscular strength and aerobic capacity. However, agility and flexibility are still poorly evaluated. In continuation, the same authors [29] highlighted that (i) the battery of fitness tests should include assessments of muscular endurance, strength, power, aerobic capacity, agility, and flexibility, which are essential occupational skills; and (ii) the need for standardisation of fitness test procedures to ensure consistency and precision when comparing results.

In 2004/2005, the Department of Physical Education and Sports—Portuguese Police Academy (Higher Institute of Police Sciences and Internal Security, 1300-663 Lisbon, Portugal) defined (and began to apply) a battery of physical fitness tests (that allow the assessment of body size, speed, agility, strength, flexibility, and aerobic capacity) to be administered to police academy cadets at the end of each academic semester of the training course for police officers (CFOP).

In accordance, to assist in tailoring training programs to different genders and age groups, which is crucial in police training, this research was conducted over the long term and involved an analysis of how various factors, such as gender, age, and 4-year training course for police officers, affect the physical fitness outcomes of Portuguese police academy cadets. Mainly, this initial approach aims (i) to confirm the impact of gender and age on the physical fitness attributes of Portuguese police academy cadets and (ii) to evaluate the effect of the 4-year training course for police officers on the physical fitness of Portuguese police academy cadets. In accordance, we believe that outcomes can be helpful for (i) the recruitment and training of cadet students and police officers, considering gender-based differences in physical fitness attributes, (ii) ongoing monitoring of the physical fitness of police officers as they age, and (iii) ensuring that future police officers are physically fit.

## 2. Materials and Methods

### 2.1. Study Design and Participants

To enter the Portuguese police academy, candidates must have completed secondary education and taken the national exams to access higher education (including candidates from the special contingent, i.e., police officers). In addition, entry requirements comprise preliminary assessments to be accepted in the police academy graduate school (i.e., physical, psychological, and medical evaluations), of which some specific fitness tests are required (see Table 1).

Candidates considered suitable are ranked, and a restricted number (~40 cadets/year) are admitted to the training course for police officers (graduation course). The academic course lasts five curricular years (master’s degree), with the fifth year dedicated to completing an internship (and a dissertation). In the first four years, the curricular structure comprises (in addition to curricular units of Social Sciences, Political Sciences, Law, and Police Sciences) two weekly Physical Education and Sports sessions.

In the 2004/2005 academic year, the Department of Physical Education and Sports defined (and started to apply) a battery of physical fitness tests to be administered at the end of each academic semester (see Section 2.2.).

**Figure 1:**
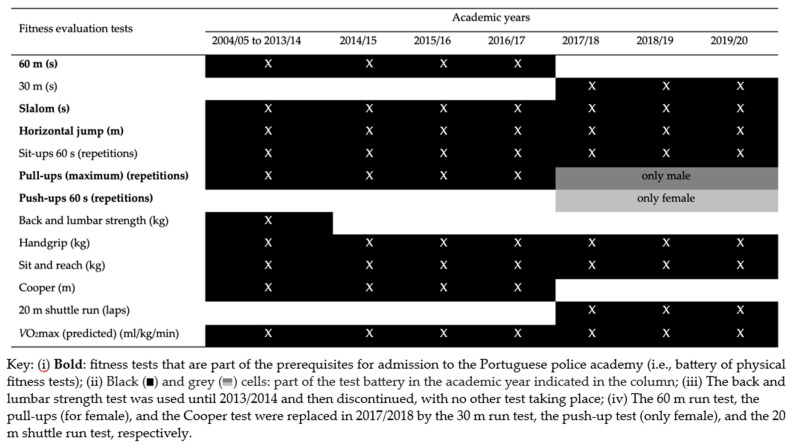
The fitness test battery adopted by the Higher Institute of Police Sciences and Internal Security (Lisbon, Portugal) between 2004/2005 and 2019/2020.

The physical fitness battery applied between the period 2004/2005 and 2019/2020 (Figure 1) allowed the assessment of speed (60 m; 30 m), agility (slalom), strength (horizontal jump, sit-ups, pull-ups, push-ups, back and lumbar strength, handgrip), flexibility (sit and reach), and aerobic capacity (Cooper test, 20 m shuttle run test, and predicted *V*O_2_max). Nevertheless, considering the evolution of the physical fitness battery, it should be noted that (i) 60 m run test, pull-ups (for females), and Cooper test, incorporated from 2004/2005 until 2016/2017, were replaced in 2017/2018 by the 30 m run test, push-ups and 20 m shuttle run test, respectively, and (ii) the back and lumbar strength test was in use until 2013/2014.

**Figure 2:**
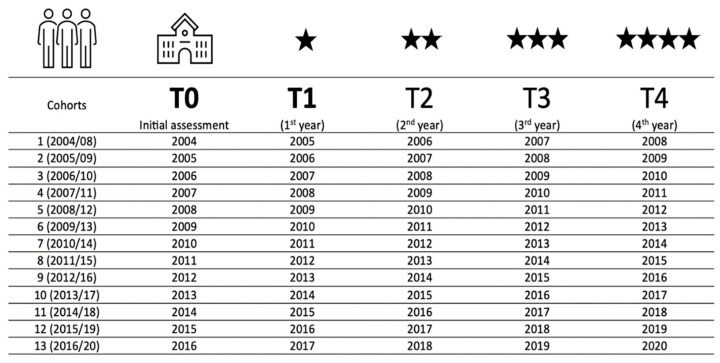
Police academy 4-year training course for police officers considered in the study (cohorts) and respective evaluation moments. Key: The last year of Cohort 13 (2016/2020) coincided with the onset of the COVID-19 pandemic.

After approval of the research project by the Higher Institute of Police Sciences and Internal Security—Lisbon—Portugal (n. ° 252/SECDE/2020, 2020-12-02), the database of police cadets’ physical fitness evaluations (from 2004/2005 to 2019/2020), carried out at the first semester of the first year (initial assessment, T0) and at the end of the second semester of each of the four academic years (T1, T2, T3, T4), was made available. This database corresponds to 13 police academy (four-year) formative training courses (13 cohorts) for graduate police officers (Figure 2).

In accordance, a total of 686 cadets (female, n = 131; male, n = 555) were considered in this longitudinal cohort study, corresponding to a total of 2578 physical fitness assessments (female, n = 509; male, n = 2069) (descriptive data in Table 1).

### 2.2. Physical Fitness Test Database

The database of police cadets’ physical fitness evaluations (from 2004/2005 to 2019/2020) comprises body size (height and body mass) and fitness evaluations.

In short, fitness variables were assessed as follows:

Sixty-metre (from 2004/2005 until 2016/2017) and thirty-metre (since 2017/2018) run tests [31]. All sprint times were recorded using electronic timing lights (Wireless Sprint System, Brower Timing Systems, Draper, UT, USA), and the time score (in seconds, s) was recorded for analysis.

**Figure 3:**
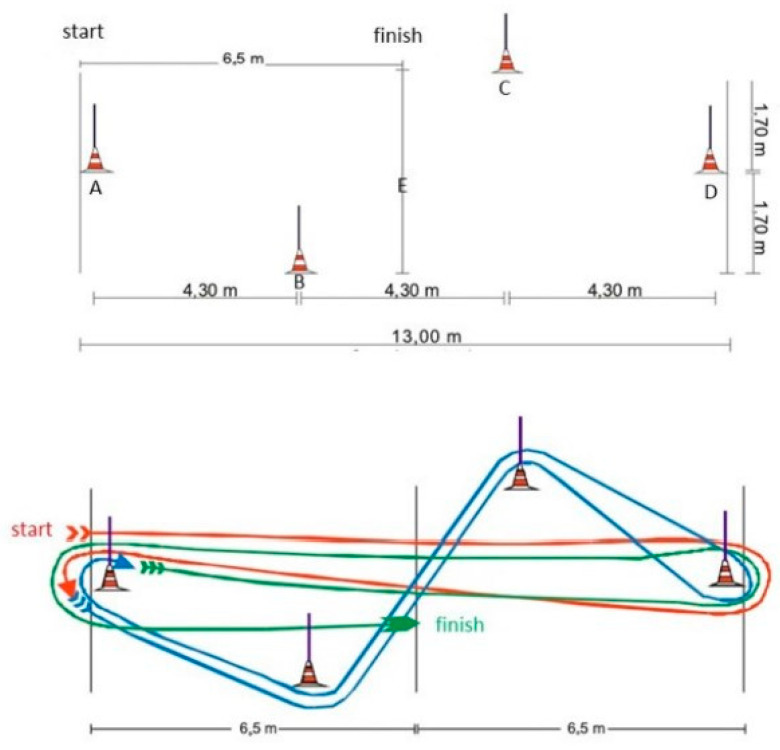
Schematic representation of the agility test adopted in 2004/2005 by the Higher Institute of Police Sciences and Internal Security—Lisbon—Portugal. Key: A–D, cones; E, finish line.

Slalom test. A standard slalom course was made up of four cones (A, B, C, and D), placed according to Figure 3, and the test (i) begins at A, then a straight line to D, and then back to A; (ii) a standard zig-zag course from A to B, C, D, then back to C, B, A; (iii) then a straight line from A to D, then back to A; and (iv) then a straight line from A to E (finish). The test stopped when the cadet (i) did not start from the stopped position, (ii) did not perform the test course in the correct order, (iii) did not circle the cone (on the outside), or (iv) touched or knocked down any cone. The final time (in seconds, s) was recorded for analysis.

Horizontal jump test [32]. The subject was asked to stand behind the starting line with parallel feet and to jump as far as possible. The jump must be performed with both feet simultaneously, using the swing of the arms and the flexion of the knees. The distance between the start line of the jump and the landing point was measured to the nearest cm.

Sit-up test in 60 s [33]. All participants start the test lying down (back on the floor), with their feet fixed and resting on the floor. The knees bent at approximately 90°, and the hands rested on the neck’s back. From the starting position, the participant must raise the body until elbows touch the knees and then return to the initial position with shoulder blades touching the floor. The number of executions (repetitions) was recorded.

Pull-up test (maximum) [34]. On a horizontal bar (placed approximately 2.50 m from the ground), the cadets were asked to stand in suspension, with hands in pronation and slightly further than shoulder-width apart, with the upper limbs in extension (elbow extended), perform the pull-ups movement, until the chin goes beyond the bar, then returning to the starting position. The number of executions (repetitions) was recorded.

Push-up test in 60 s [17] (only female cadets; since 2017/2018). Start in a plank position, supporting feet and hands on the ground, with hands approximately shoulder-width apart, with fingers pointing forward and the back sealed. One repetition was counted whenever, after flexing the upper limbs, the subject touched the wooden plate (T-shaped) with the chest and returned to the starting position. The number of executions (repetitions) was recorded.

Back and lumbar isometric strength test [35]. An external force is applied to a handle, attached to an adjustable chain, a steel spring compresses, and a pointer moves (the dial ranges from 0 to 300 kg in 10 kg increments). The subject stood on the base of the BLC dynamometer (Baseline, New York, NY, USA) with extended knees, and the chain length was adjusted (the handle was positioned at the height of the intra-articular space of the knee joint). For the test, cadets (i) stood on the base, with knees and hips flexed slightly while the lower back had to maintain an appropriate lordotic curve, and (ii) gradually increased the pull and reached the maximal force in 3 s and maximal strength (in kg) was recorded.

Handgrip strength test [36]. Participants performed the test twice with each hand (using a dynamometer: model J00105—Sammons Preston, Bolingbrook, Illinois, or Takei Physical Fitness Test, TKK 5001, GRIP–A, Tokyo, Japan). The sum of the best results achieved by both hands (in kg) was calculated and recorded.

Sit and reach test [27,37]. Seated barefoot on the floor with legs straight ahead and their feet placed with the soles flat against the sit and reach box (Acuflex I, by Novel Products Inc. P.O. Box 408, Rockton, IL, USA). A yardstick is at the top of the box, and the zero point is at 38 cm. The cadet should slowly reach forward with extended arms, placing one hand on top of the other, facing palms down, as far as possible, holding the final position for approximately 3 s and the distance (in cm) was recorded.

Cooper test (from 2004/2005 until 2016/2017) [38]. This test was performed on a 400 m synthetic athletic track, and cadets covered the maximum possible distance for 12 min. The distance covered (in m) was recorded and used to predict *V*O_2_max (*V*O_2_max, in mL/kg/min = distance (in m) − 504/45).

A 20 m shuttle run test (since 2017/2018) [39]. Participants ran back and forth between two lines, 20 m apart at 8.5 km/h, with the speed increasing by 0.5 km/h/min. The test continued until participants reached exhaustion or could not complete the laps twice continuously within the required time limit. The number of shuttles at the final stage was recorded (laps) and used to predict *V*O_2_max (in mL/kg/min) by applying the equation proposed by Ramsbottom et al. [40].

### 2.3. Statistical Analysis

Descriptive and inferential statistical analyses were performed with the Statistical Package for the Social Sciences computer program (v.25, SPSS Inc., Chicago, IL, USA). The effects whose *p*-value was less than or equal to 0.05 were considered statistically significant.

The significance of the difference between the mean values of the physical fitness variables in the female vs. male cadets was assessed with the *t*-Student test (the assumptions of this statistical method, namely the normality of distributions and the homogeneity of variances in the two groups, were evaluated, respectively, with the Kolmogorov–Smirnov test with Lilliefors correction and the Levene’s test). The results are presented as mean (M), standard deviation (SD) and standard error of the mean (SEM).

Linear Mixed Models were also used to assess whether age class (<20 years; 20 to 29 years; >29 years) significantly affected performance on physical fitness tests, and the results of Bonferroni’s univariate and multiple comparisons tests are presented. Finally, the performance percentiles (P5, P10, P20, P25, P30, P40, P50, P60, P70, P75, P80, P90, and P95) of female and male cadets on the physical fitness tests were calculated overall and by age classes (≤29 years; >29 years).

Mixed linear models assessed changes between the five survey time points. Physical fitness variables acted as dependent variables, while time (T0, T1, T2, T3, T4) was the independent variable (fixed factor). The repeated covariance type was set to the composite symmetry matrix (CS), and the model fitting method was restricted to maximum likelihood (REML). Multiple mean comparisons with Bonferroni correction were performed to determine the differences between T0 (initial assessment) and the evaluation at the end of each academic year (T1, T2, T3, T4). Values reported (difference in means; SEM, standard error of the mean; 95%CI, 95% confidence interval) are based on estimated marginal means. Effects whose *p* ≤ 0.05 were considered statistically significant, and effect sizes were calculated by subtracting the estimated marginal means at T1, T2, T3, and T4 from T0 and dividing the result by the standard deviation of T0. Effect size thresholds (used to assess the magnitude of the difference in means) were interpreted as (i) small, ≤0.19; (ii) medium, 0.20–0.49; (iii) high, 0.50–0.79; and (iv) very high, ≥0.80 [41].

## 3. Results

### 3.1. Differences between Genders

Female cadets are, on average, significantly younger, shorter, and lighter, less fast (60 m and 30 m) and agile (slalom), less strong (horizontal jump, sit-ups, pull-ups, back and lumbar strength, handgrip), and perform worse in aerobic capacity assessments (Cooper test and 20 m shuttle run test; *V*O_2_max) than male cadets. However, female cadets perform better than male cadets in the flexibility assessment (sit and reach test). The results are shown in Table 2.

### 3.2. Differences between Age Groups

Significant effects of age on body mass (both sexes) are observed, and younger cadets (<20 years) are the lightest, and those >29 years are the heaviest (significantly different from the two younger age classes).

A significant effect of age on speed (60 m and slalom), strength (horizontal jump, sit-ups, and handgrip), and Cooper test are observed on female cadets. Cadets > 29 years are significantly slower, jump less, perform fewer sit-ups, and perform worse on the Cooper test. However, they have significantly higher values in handgrip strength.

In male cadets, significant differences are observed between age groups in all fitness attributes (except in 30 m), and (i) the older age class (>29 years) are slower (60 m and slalom), jump less, perform fewer repetitions in the sit-up test, have worse performances in the sit and reach and aerobic capacity assessment tests; and (ii) the younger age class (<20 years) perform fewer repetitions on the pull-up test and have less back and lumbar strength and handgrip strength.

The results are presented in Table 3.

Complementarily, the fitness percentiles were calculated, and given the significant differences between the age class of >29 years and younger age classes, the percentiles for each of the age groups ≤29 years and >29 years were calculated (see Table 4).

### 3.3. Effect of the 4-Year Training Program

Regarding the differences between T0 and the evaluation at the end of each academic year (T1, T2, T3, T4), results showed the significant effect of training course for police officers in female cadets’ speed (60 m and slalom) and strength tests (horizontal jump, sit-ups and back and lumbar strength), i.e., at the end of four years, they are significantly faster (medium effect size: 60 m, Δ = −0.32 s; slalom, Δ = −0.78 s), jump further (medium effect size: Δ = +4 cm), have more abdominal strength endurance (medium effect size: Δ = +2.6 repetitions), more back and lumbar strength (very high effect size: Δ = +89.8 kg), and superior performance on Cooper test (medium effect size: Δ = +89.8 kg).

Complementarily, the study of body mass differences between the initial assessment (T0) and the evaluation at the end of each academic year (T1, T2, T3, T4) showed highly significant differences in males from T2 to T4 (medium effect size), with an average increase of 1.6 kg, 2.0 kg, and 3.3 kg, respectively. Furthermore, significant training effects are observed in performance, i.e., on speed (all), strength (all), flexibility, and aerobic capacity (Cooper test and predicted *V*O_2_max). In addition, after a 4-year training course for police officers (T4), male cadets are significantly faster (very high effect size: 60 m, Δ = −0.23 s; 30 m, Δ = −0.15 s; slalom, Δ = −0.91 s), jump further (medium effect size: Δ = +8 cm), complete more repetitions in the sit-up test (very high effect size: Δ = +4.9 repetitions), complete more repetitions in the pull-up test (medium effect size: Δ = +2.5 repetitions), have superior back and lumbar strength (very high effect size: Δ = +92.1 kg) and superior handgrip strength (medium effect size: Δ = +8.6 kg). Nevertheless, they present a lower aerobic capacity when compared to T0 (medium effect size: Cooper test, Δ = −74.8 m; *V*O_2_max, Δ = −1.3 mL/kg/min).

**Figure 4:**
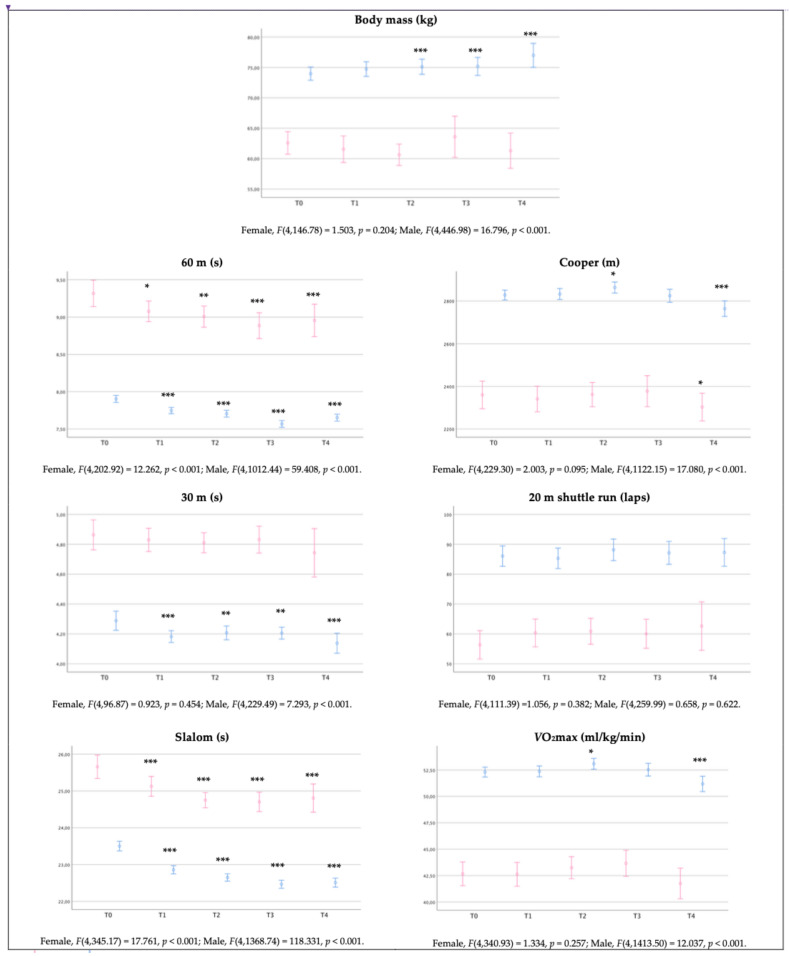
Representation (mean ± confidence interval) of Portuguese police academy cadets’ body mass, speed, agility and aerobic capacity outcomes at the initial assessment and the end of the second semester of each of the four academic years. Key: 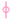, female; 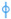, male; T0, initial assessment; T1, first year; T2, second year; T3, third year; T4, fourth year; statistics (difference from T0)—***, *p* < 0.001 or **, *p* < 0.01 or *, *p* < 0.05.

**Figure 5:**
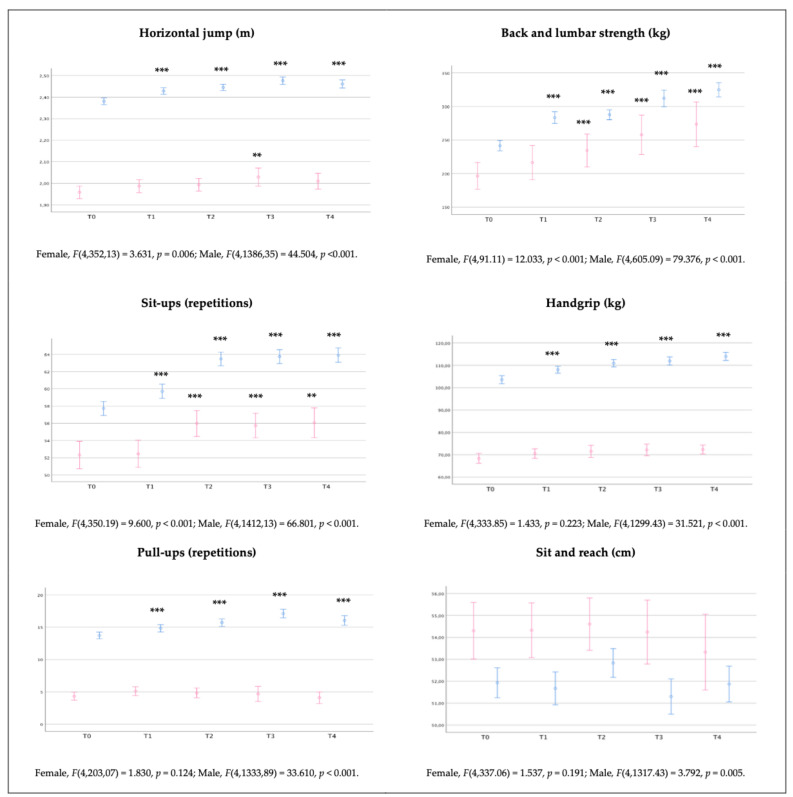
Representation (mean ± confidence interval) of Portuguese police academy cadets’ strength and flexibility outcomes at the initial assessment and the end of the second semester of each of the four academic years. Key: 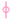, female; 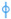, male; T0, initial assessment; T1, first year; T2, second year; T3, third year; T4, fourth year; Statistics (difference from T0)—***, *p* < 0.001 or **, *p* < 0.01.

The results are presented in Table 5 and graphically in Figure 4 and Figure 5.

## 4. Discussion

The research was conducted over the long term. It involved an analysis of how various factors, such as gender, age, and four years of the training course for police officers, affect the physical fitness of Portuguese police academy cadets.

Both men and women were included in the study, allowing for a more comprehensive understanding of this issue. This study (i) showed that male cadets are, on average, 24 years old and have a height of 1.78 m and a body weight of 74.9 kg, while female cadets are, on average, are 23 years old and have a height of 1.67 m and a body weight of 61.9 kg; and (ii) confirms that male cadets have significantly better performance in most fitness tests (but female cadets have superior performance in the flexibility test). This significant finding could have implications for the recruitment and training of police officers, considering gender-based differences in physical fitness.

Regarding gender differences, our study corroborates the established narrative that male cadets generally outperform female cadets in most physical fitness tests, except for flexibility, where female cadets excel. This divergence in performance is not merely a reflection of physical disparities but also underscores the potential for optimising training regimes. It suggests a nuanced approach to training, where individual strengths and weaknesses are acknowledged and addressed. This approach could lead to more effective training outcomes, enhancing overall fitness and operational readiness.

The pattern presented is also consistent with (i) Sharp et al. [42], who compared physical performance between male and female applicants in the U.S. Army between 1978 and 1998 and observed better indicative physical fitness performance scores in men when compared with women in the same year; (ii) Lagestad and Tillaar [5], who found significant differences between male and female performances on three of the four physical fitness tests (the only exception was pull-ups, where females performed a modified lift, namely inverted rows); and (ii) Maupin et al. [10], whose study indicated that male cadets performed significantly better than female cadets in all physical fitness tests. This pattern aligns with earlier findings from the U.S. Army and other law enforcement settings, reinforcing the need for gender-specific training strategies to enhance physical performance across all fitness domains.

Regarding age classes, it was observed that (i) female cadets aged >29 years are significantly slower, jump less, have lower abdominal strength endurance and aerobic capacity, and show significantly higher values in handgrip strength than younger female cadets; and (ii) male cadets aged >29 years are significantly slower, jump less, have lower abdominal strength endurance, flexibility, and aerobic capacity than male cadets from the other two age classes. Nevertheless, male cadets aged <20 years appear to have significantly less strength, i.e., perform fewer repetitions on the pull-up test and have less back, lumbar, and handgrip strength.

It should be noted that (i) explosive and maximum strength decrease with age, with the decline of the former being more accentuated [43], i.e., maximal strength is reached around the age of 30 years and remains stable until the fifth decade of life when the decline begins (i.e., 50 to 70 years, −15% per decade; after 70 years, −30% every ten years) [44]; (ii) flexibility begins a decline during adulthood [45]; and (iii) the age-related decline in *V*O_2_max is approximately 5–10% per decade, with the value being lower or higher depending on the level of physical activity [46].

The Portuguese police academy’s 4-year training course for police officers reaches a minimum of 17 years of entry age and a maximum of 39 years (the maximum entry age for agents and chiefs for the first academic year is 35 years). Given the range of ages within the Portuguese police academy, these variations should be considered as these declines apply to the attributes to be assessed by the current battery of fitness tests.

Nevertheless, and contrary to the above and the results of this study, it is noteworthy that Maupin et al. [10] observed (i) no change or (on specific tests) an improvement in performance from age 20–29 to 30–39 years (regardless of gender), and (ii) a significant decrease from age 40 years onwards. A recent study by Oliveira et al. [47] further supports this notion, highlighting that the cadets’ physical fitness improved with age, particularly peaking in the 22–23 age group, and that their training regimen was conducive primarily to meeting the fitness benchmarks required, albeit with some suggested modifications for optimised training outcomes. This observation also may reinforce that, although the loss of physical fitness is inevitable because of advancing age, this decrease can be countered at the ages considered in this work, i.e., by reducing the loss of fitness attributes, maintaining them, or even improving them.

In sum, the study revealed that as individuals age, both men and women tend to experience a decline in physical fitness, although there were exceptions, such as handgrip strength and back and lumbar strength. In other words, the study highlighted notable age-related differences in physical performance among the cadets (cadets > 29 years exhibited lower aerobic capacity, flexibility, and abdominal strength endurance than their younger counterparts and showed higher values in handgrip strength). Notably, the age-related decline in *V*O_2_max, flexibility, and explosive and maximum strength, as discussed in previous literature, underscores the necessity to account for age while designing training programs, especially given the broad age range (17 to 39 years) of cadets enrolled in the Portuguese police academy. Moreover, our results suggest the need for ongoing monitoring of the physical fitness of police officers as they age.

Furthermore, the age-related findings in our study extend beyond the predictable narrative of physical decline with advancing age. While it is well-documented that physical fitness attributes like *V*O_2_max, flexibility, and explosive strength generally diminish over time, our observations suggest a more intricate pattern. The age group of 22–23 years showed notable improvements [47], indicating that the training regimen’s effectiveness peaks at this age. This insight is crucial for tailoring training programs to maximise the physical capabilities of cadets at different ages, ensuring they are not only fit for service upon graduation but also equipped to maintain their fitness throughout their careers.

Nevertheless, although body composition is a component of physical fitness present in the vast majority of the reviewed literature and one of the essential components in a physical fitness test battery [26], it is not found in the present combination of tests used by the Portuguese police academy, which demonstrates a gap in the monitoring of the physical fitness of cadets, since having an above average weight/obesity is relatively common in the army and security forces [48].

This is even more relevant since that body weight and fat mass increase with ageing, but total fat-free mass and its constituents (e.g., skeletal muscle mass, total body water and bone mineral mass) gradually decrease (approximately 16% between the ages of 25 and 70 years in men and women, at a rate of ~0.16 kg/year). Knowing that skeletal muscle is responsible for more than half (~55%) of the total fat-free mass, the decrease in skeletal muscle mass is understandable. According to specific literature, an increase in relative fat mass (%FM) appears to be associated with a decrease in performance and physical fitness [18,49,50], i.e., higher %FM results in lower cardiorespiratory capacity, lower dynamic strength and lower flexibility [49]. Therefore, a decrease in %FM and an increase in lean body mass can positively affect physical performance [18].

Moreover, the findings of this long-term study underscore the importance of a tailored approach in the physical training of Portuguese police academy cadets. The observed variations in physical performance across different genders and age groups over the 4-year training course emphasise the need for an individualised training regimen to meet the diverse needs and ensure the optimal fitness levels of all cadets.

This observation also emphasises the relevance of studying the effect of the 4-year training course for police officers on cadet students’ physical fitness attributes.

In this context, considering the minimum age for admission to the 4-year training course for police officers (entering adulthood), cadets’ growth and height are expected to stagnate. Nevertheless, in an initial approach, it is observed that the body mass of male cadets (in contrast to what is observed in females) increased significantly from the first (T1) to the last year (T4) of the training course for police officers (Δ = +3 kg, i.e., from 74.0 to 77.0 kg). Nevertheless, regarding the effect of the 4-year of training course for police officers on the physical fitness attributes of police academy cadets (i.e., from T0 to T4), were observed: (i) improvements in speed (in 60 m and 30 m run tests), agility (significant only in male cadets), and in all strength evaluations (female, only in the back and lumbar strength; male, all strength tests), and the 20 m shuttle run test (not significant); and (ii) decreases in the flexibility (not significant), and Cooper test and *V*O_2_max (predicted) for both sexes (significant in males).

These results suggested a superior investment in strength and speed training (two mutually beneficial areas) throughout the 4-year training course for police officers. At the same time, flexibility was maintained, and aerobic capacity decreased slightly in male cadets. Nevertheless, this decline in aerobic capacity may be because (i) in the 4th year of the training course for police officers, cadets are “finalists” and do not need to invest much in this test (which is the most “hard/difficult” and has the same value/weight in the battery of fitness assessment as the other tests); and (ii) little value/weight of classification in the curricular unit (i.e., physical education and sports) in the final grade of the 4-year training course for police officers. On the other hand, the evidence that speed directly benefits from strength training in non-competitive athletes [45] may have motivated cadets to invest more in training these fitness attributes at the expense of aerobic capacity. Indeed, this was also observed by Lagestad and Tillaar [5].

The 4-year training course positively impacted the cadets’ physical fitness attributes, with improved speed, agility, and strength evaluations. Conversely, a slight decline in aerobic capacity was observed among male cadets, possibly due to a reduced emphasis on aerobic training in the final year of the course. The evidence suggesting a direct benefit of strength training on speed, as highlighted by Lagestad and Tillaar [5], reinforces the potential advantage of a balanced training regimen focusing on strength and aerobic conditioning.

In general, this study suggested that performance on specific fitness tests is improved/maintained during the Portuguese police academy (4-year) training course for police officers, suggesting that the curriculum responds to one of the police academy’s missions (i.e., prepare recruits for their future professional careers), as the practice of physical activity, physical exercise and consequent development of physical fitness not only (i) prepare cadets to deal with the occupational impact of the profession, (ii) but can also benefit them in physical fitness terms throughout their career [10]. This improvement may lead to police officers with a lower risk of injury, more excellent physical fitness, psychological well-being, and resilience in the long term [6,25].

Interestingly, the increase in body mass among male cadets and the maintenance/improvement of specific fitness attributes throughout the 4-year training course indicate that the current curriculum effectively prepares cadets for the physical demands of their future professional careers. This improvement might translate to lower injury risks, enhanced physical fitness, psychological well-being, and long-term resilience among the police officers, which aligns with the mission of the police academy.

It is essential to highlight that our study’s value lies not in merely outlining these differences but in providing a database for the foundation for re-evaluating (and potentially reforming) training methodologies at the police academy by delving into how age and gender impact physical fitness. We pave the way for more personalised and effective training strategies. These strategies could be pivotal in preparing cadets for the diverse challenges of policing, ensuring they are physically capable, adaptable, and resilient in the face of the evolving demands of law enforcement.

Although the inclusion (in the study) of physical fitness tests recently introduced in the test battery conditioned the number of observations available for analysis, it seems that this work brings with it the advantage of making known (i) the physical fitness profile of Portuguese police academy cadets and (ii) the effect that the 4-year of the training course for police officers has on the enunciated profile. It seems relevant to highlight that maximal tests performed in the laboratory are more effective for quantifying *V*O_2_max than the Cooper (12-min run test) or 20 m shuttle run tests. However, the fact that field tests make it possible to evaluate several individuals simultaneously with minimal equipment justifies their selection. On the other hand, the assessment of musculoskeletal fitness considers evaluative tests of muscular strength endurance, maximum strength, and flexibility, with a balanced distribution between upper (e.g., push-ups; abdominal strength endurance; handgrip) and lower (e.g., horizontal jump, sit and reach), emphasising that the inclusion of a flexibility test is a positive because his dimension is not often assessed in military and police environments [26]. Finally, the inclusion of fitness assessment seems (i) to have advantages since work efficiency seems to be related to tests of coordination and speed, showing the individual’s ability to be efficient [43], and (ii) to be a predictor of injury onset [26] (e.g., agility and balance training are effective in preventing and reducing injuries [51]).

Despite the observed positive trends, the absence of body composition assessment in the current fitness test battery presents a gap in monitoring cadet physical fitness. The prevalence of overweight/obesity among military and security forces and its associated health and economic challenges (with overweight/obese military personnel being at a higher risk of taking sick leave, especially long-term sick leave, and obesity being associated with productivity losses) as discussed by Neovius et al. [52], underlines the importance of incorporating body composition monitoring to provide a holistic evaluation of cadet physical fitness. Such inclusion could enhance the ability to perform physical tasks and improve cardiorespiratory fitness, thereby contributing to the overall effectiveness and well-being of the police force. In sum, monitoring the evolution and improvement of body composition indicators may increase the ability to perform other physical tasks successfully.

## 5. Conclusions

The research was conducted over the long term and involved an analysis of how various factors, such as gender, age, and police academy training, affect the physical fitness of Portuguese police academy cadets. Both men and women were included in the study, allowing for a more comprehensive understanding of this issue.

Several important conclusions were drawn from the study.

- The clear difference between genders (with men performing better in physical fitness tests than women) could have implications for the recruitment and training of police officers, considering gender-based differences in physical fitness attributes.
- As individuals age, both men and women tend to experience a decline in physical fitness (however, there are exceptions, such as handgrip strength and back and lumbar strength), which suggests the need for ongoing monitoring of the physical fitness of police officers as they age.
- The 4-year training course for police officers effectively maintains or improves the physical fitness attributes of Portuguese police academy cadets, and this positive outcome can help ensure that future police officers are physically fit.

In conclusion, this initial work (i) provides valuable insights into the impact of various factors on the physical fitness of Portuguese police academy cadets; (ii) seems essential, with practical implications for recruitment, training, and the ongoing development of police officers; and (iii) can also assist in tailoring training programs to different age groups and genders, which is crucial in police training.

## Figures and Tables

**Table 1:** Fitness requirements (cut-off values) for police academy candidates.

Fitness Tests	2004/2005 to 2009/2010 (*)		2010/2011 to 2019/2020 (**)	
	Female	Male	Female	Male
60 m (s)	-	-	9.70	8.80
100 m (s)	16.4	13.9	-	-
Slalom (s)			25.2	24.0
Wall jump (m)	0.80	1.00	0.80	1.00
Sargent jump test (m)	0.35	0.45	-	-
Horizontal jump (m)	1.80	2.20	1.80	2.20
Pull-ups (repetitions)	2	5	-	3
Push-ups (repetitions)	-	-	10	-
Sit-ups in 45 s (repetitions)	25	30	25	30
1000 m (minute: seconds)	4.35	3.40	4.35	3.40

Key: Published in national legislation, i.e., (*), Service Order. n. ° 89/93—Part II, of 22 March; (**), Republic Diary n. ° 85, II Series of 3 May, Notice n. ° (8682/2010) and Ordinance n. ° 230/2010 of 26 April.

**Table 2:** Descriptive statistics of physical fitness of police academy cadets by gender.

	Female				Male				*t*-Test				
									*t*	*df*	*Sig.*	95% CI	
	n	M	SD	SEM	n	M	SD	SEM				Lower	Higher
Age (years)	304	23.31	4.75	0.27	1332	24.12	5.07	0.14	−2.521	1634	**0.012**	−1.428	−0.178
Height (m)	473	1.67	0.05	0.00	1753	1.78	0.07	0.00	−39.405	970.46	**<0.001**	−0.115	−0.104
Body mass (kg)	253	61.87	7.97	0.50	846	74.92	8.69	0.30	−22.361	448.19	**<0.001**	−14.199	−11.905
60 m (s)	295	9.06	0.65	0.04	1411	7.73	0.40	0.01	33.833	342.58	**<0.001**	1.258	1.413
30 m (s)	149	4.82	0.24	0.02	322	4.21	0.20	0.01	27.269	248.16	**<0.001**	0.573	0.662
Slalom (s)	477	25.06	1.46	0.07	1894	22.85	1.23	0.03	33.710	2369	**<0.001**	2.079	2.335
Horizontal jump (m)	479	1.99	0.16	0.01	1908	2.43	0.16	0.00	−52.878	2385	**<0.001**	−0.458	−0.426
Sit-ups (repetitions)	473	54.23	7.97	0.37	1890	61.44	8.49	0.20	−17.365	762.94	**<0.001**	−8.023	−6.393
Pull-ups (repetitions)	284	4.65	2.96	0.18	1848	15.34	5.99	0.14	−47.668	708.00	**<0.001**	−11.128	−10.248
Push-ups (repetitions)	149	27.04	6.50	0.53	-	-			-	-	-	-	-
Back and lumbar strength (kg)	148	226.36	71.91	5.91	880	283.27	67.16	2.26	−9.439	1026	**<0.001**	−68.744	−45.082
Handgrip (kg)	448	70.79	11.55	0.55	1784	109.43	16.90	0.40	−57.105	985.35	**<0.001**	−39.969	−37.313
Sit and reach (cm)	462	54.24	6.52	0.30	1811	51.95	7.13	0.17	6.608	767.39	**<0.001**	1.610	2.971
Cooper (m)	314	2349.78	254.40	14.36	1540	2826.67	248.95	6.34	−30.822	1852	**<0.001**	−507.24	−446.55
20 m shuttle run (laps)	151	59.70	13.37	1.09	367	86.76	16.10	0.84	−18.232	516	**<0.001**	−29.984	−24.151
Predicted *V*O_2_max (mL/kg/min)	465	42.85	5.74	0.27	1907	52.36	5.47	0.13	−33.262	2370	**<0.001**	−10.070	−8.949

Key: M, mean; SD, standard deviation; SEM, standard error of the mean; CI, confidence interval.

**Table 3:** Descriptive statistics of physical fitness of police academy cadets by age classes.

	Total			Age Classes									Statistics					
				<20 Years (1)			20–29 Years (2)			>29 Years (3)			Univariate Test			Bonferroni Test		
	n	M	SD	n	M	SD	n	M	SD	n	M	SD	*F*	*df*	*Sig.*	1–2	1–3	2–3
Female cadets																		
Body mass (kg)	167	61.50	8.30	49	59.17	6.51	89	60.18	6.44	29	69.49	11.17	20.302	(2.164)	**<0.001**	1.000	**<0.001**	**<0.001**
60 m (s)	167	9.07	0.73	33	9.00	0.46	111	8.91	0.53	23	9.95	1.14	25.830	(2.164)	**<0.001**	1.000	**<0.001**	**<0.001**
30 m (s)	90	4.83	0.23	22	4.83	0.18	52	4.82	0.25	16	4.88	0.22	0.429	(2.87)	0.653	1.000	1.000	1.000
Slalom (s)	285	25.26	1.43	65	25.04	1.13	179	25.08	1.22	41	26.42	2.04	17.788	(2.282)	**<0.001**	1.000	**<0.001**	**<0.001**
Horizontal jump (m)	284	2.00	0.18	65	1.99	0.16	177	2.02	0.16	42	1.90	0.23	8.930	(2.281)	**<0.001**	0.918	**0.012**	**<0.001**
Sit-ups (repetitions)	277	55.16	8.03	63	55.37	8.07	173	56.03	7.18	41	51.15	10.12	6.407	(2.274)	**0.002**	1.000	**0.024**	**0.001**
Pull-ups (repetitions)	171	4.97	2.79	37	4.89	2.02	116	5.14	3.05	18	4.06	2.24	1.196	(2.168)	0.305	1.000	0.892	0.380
Push-ups (repetitions)	91	27.64	6.78	23	28.48	7.65	53	27.81	6.30	15	25.73	7.12	0.782	(2.88)	0.460	1.000	0.680	0.896
Back and lumbar strength (kg)	113	218.24	74.99	27	198.86	66.06	74	220.87	74.03	12	245.69	93.73	1.775	(2.110)	0.174	0.575	0.218	0.860
Handgrip (kg)	269	71.39	11.38	63	69.59	10.76	166	70.52	11.43	40	77.86	10.10	8.143	(2.266)	**<0.001**	1.000	**0.001**	**0.001**
Sit and reach (cm)	270	54.28	6.51	60	54.96	5.43	170	53.64	7.06	40	55.97	5.10	2.522	(2.267)	0.082	0.532	1.000	0.124
Cooper (m)	180	2340.68	247.10	37	2347.49	273.49	119	2376.30	201.60	24	2153.58	326.22	8.843	(2.177)	**<0.001**	1.000	**0.006**	**<0.001**
20 m shuttle run (laps)	92	61.08	13.65	24	60.29	12.79	52	60.92	12.77	16	62.75	17.89	0.160	(2.89)	0.852	1.000	1.000	1.000
Predicted *V*O_2_max (mL/kg/min)	272	42.95	5.76	61	43.27	5.95	171	43.27	4.84	40	41.08	8.37	2.498	(2.269)	0.084	1.000	0.184	0.090
Male cadets																		
Body mass (kg)	608	75.63	8.56	127	72.61	8.09	377	75.74	8.50	104	78.90	8.15	16.310	(2.605)	**<0.001**	**0.001**	**<0.001**	**0.002**
60 m (s)	891	7.73	0.41	169	7.71	0.38	574	7.69	0.39	148	7.90	0.47	16.253	(888.2)	**<0.001**	1.000	**<0.001**	**<0.001**
30 m (s)	200	4.23	0.20	28	4.26	0.24	128	4.21	0.20	44	4.27	0.18	2.290	(197.2)	0.104	0.524	1.000	0.175
Slalom (s)	1218	22.93	1.22	245	22.87	1.04	776	22.83	1.20	197	23.42	1.35	19.490	(1215.2)	**<0.001**	1.000	**<0.001**	**<0.001**
Horizontal jump (m)	1239	2.43	0.16	246	2.41	0.16	793	2.45	0.17	200	2.39	0.15	11.314	(1236.2)	**<0.001**	**0.013**	0.468	**<0.001**
Sit-ups (repetitions)	1222	62.65	8.18	232	61.79	8.12	785	63.31	8.12	205	61.10	8.22	7.627	(1219.2)	**0.001**	**0.037**	1.000	**0.002**
Pull-ups (repetitions)	1177	15.09	5.93	230	13.65	5.12	747	15.71	5.98	200	14.47	6.27	12.136	(1174.2)	**<0.001**	**<0.001**	0.457	**0.024**
Back and lumbar strength (kg)	667	278.38	65.91	141	249.52	58.54	432	282.37	66.51	94	303.30	59.03	22.381	(664.2)	**<0.001**	**<0.001**	**<0.001**	**0.012**
Handgrip (kg)	1146	109.73	17.36	206	103.56	16.24	743	110.10	17.61	197	114.79	15.65	22.348	(1143.2)	**<0.001**	**<0.001**	**<0.001**	**0.002**
Sit and reach (cm)	1154	51.63	7.04	215	52.71	6.43	742	51.62	7.01	197	50.48	7.60	5.223	(1151.2)	**0.006**	0.132	**0.004**	0.128
Cooper (m)	996	2823.26	256.36	196	2839.83	243.36	648	2830.67	256.27	152	2770.32	267.90	3.944	(993.2)	**0.020**	1.000	**0.036**	**0.027**
20 m shuttle run (laps)	232	87.62	16.55	34	87.94	16.53	145	89.54	16.37	53	82.15	16.15	3.971	(229.2)	**0.020**	1.000	0.325	**0.016**
Predicted *V*O_2_max (mL/kg/min)	1228	52.35	5.65	230	52.55	5.37	793	52.58	5.67	205	51.19	5.75	5.178	(1225.,2)	**0.006**	1.000	**0.036**	**0.005**

Key: M, mean; SD, standard deviation.

**Table 4:** Age-specific percentile values (P_5_ to P_95_) for fitness tests for police academy cadets.

	Female																Male															
	n	M	SD	P_5_	P_10_	P_20_	P_25_	P_30_	P_40_	P_50_	P_60_	P_70_	P_75_	P_80_	P_90_	P_95_	n	Mean	SD	P_5_	P_10_	P_20_	P_25_	P_30_	P_40_	P_50_	P_60_	P_70_	P_75_	P_80_	P_90_	P_95_
≤29 years																																
60 m (s)	144	8.93	0.52	8.2	8.3	8.5	8.5	8.7	8.8	8.9	9.1	9.2	9.3	9.4	9.6	9.9	148	7.90	0.47	7.2	7.3	7.5	7.5	7.6	7.7	7.9	8.0	8.1	8.2	8.3	8.5	8.7
30 m (s)	74	4.82	0.23	4.5	4.5	4.6	4.7	4.7	4.8	4.8	4.9	4.9	5.0	5.0	5.1	5.2	44	4.27	0.18	4.0	4.1	4.1	4.1	4.1	4.2	4.2	4.3	4.4	4.4	4.5	4.6	4.6
Slalom (s)	244	25.07	1.19	23.3	23.7	24.1	24.4	24.5	24.8	25.0	25.2	25.5	25.7	25.9	26.5	27.2	197	23.42	1.35	21.5	21.8	22.2	22.4	22.7	23.0	23.2	23.6	24.0	24.2	24.5	25.0	26.2
Horizontal jump (m)	242	2.01	0.16	1.80	1.83	1.88	1.90	1.92	1.95	2.00	2.05	2.08	2.10	2.15	2.21	2.27	200	2.39	0.15	2.10	2.20	2.28	2.30	2.33	2.38	2.41	2.45	2.47	2.49	2.51	2.56	2.58
Sit-ups (repetitions)	236	55.86	7.42	43	46	50	51	52	54	56	59	60	61	62	66	68	205	61.10	8.22	46	49	55	55	57	59	62	64	66	68	69	71	73
Pull-ups (repetitions)	153	5.08	2.83	2	2	3	3	3	4	4	6	6	6	7	9	10	200	14.47	6.27	6	7	9	10	10	13	13	14	16	19	21	25	25
Push-ups (repetitions)	76	28.01	6.69	13	15	24	27	30	30	30	30	30	30	31	34	36																
Back and lumbar strength (kg)	101	214.98	72.32	100.4	111.2	139.6	157.8	176.6	200.0	218.9	231.4	251.0	262.7	283.61	327.9	342.9	94	303.30	59.03	198.4	219.5	254.8	259.6	275.0	298.6	300.0	310.0	332.2	341.9	356.8	386.8	402.8
Handgrip (kg)	229	70.26	11.24	53.3	57.0	61.0	63.0	64.0	66.5	69.3	72.0	75.0	76.0	78.0	83.0	90.0	197	114.79	15.65	91.1	97.8	101.7	103.3	105.0	110.0	113.4	117.2	121.8	124.1	127.4	137.0	143.3
Sit and reach (cm)	230	53.99	6.69	45	47	50	51	51	52	54	55	57	58	59	62	64	197	50.48	7.60	40	41	44	45	46	48	50	53	55	57	57	61.0	62.1
Cooper (m)	156	2369.47	220.15	2100	2143	2200	2200	2250	2300	2350	2400	2497	2543	2600	2652	2700	152	2770.32	267.90	2400	2400	2550	2600	2600	2700	2728	2800	2900	2950	3000	3100	3300
20 m shuttle run (laps)	76	60.72	12.70	39.70	44	48	51	53	58	60	64	69	71	74	77	83	53	82.15	16.15	56	65	69	70	72	76	80	83	87	90	93	107	117
Predicted *V*O_2_max (mL/kg/min)	232	43.27	5.14	35.7	37.3	38.6	39.4	40.1	41.6	42.4	44.6	47.0	47.6	47.6	50.6	50.6	205	51.19	5.75	42.4	44.6	46.8	46.8	47.71	49.1	50.6	51.6	53.6	54.7	55.8	59.3	62.5
>29 years																																
60 m (s)	23	9.95	1.14	8.1	8.7	9.0	9.2	9.2	9.3	9.7	10.1	10.6	10.6	10.9	12.0	12.3	743	7.70	0.39	7.1	7.2	7.4	7.4	7.5	7.6	7.7	7.8	7.9	8.0	8.0	8.2	8.3
30 m (s)	16	4.88	0.22	4.5	4.6	4.7	4.8	4.8	4.8	4.9	4.9	4.9	5.1	5.1	5.3	-	156	4.22	0.21	3.9	4.0	4.0	4.1	4.1	4.2	4.2	4.2	4.3	4.3	4.4	4.5	4.6
Slalom (s)	41	26.42	2.04	23.7	24.1	24.7	24.8	25.0	25.4	25.9	27.1	27.6	27.8	28.0	29.1	30.3	1021	22.84	1.16	21.1	21.5	21.8	22.0	22.2	22.4	22.7	23.0	23.4	23.6	23.8	24.3	24.8
Horizontal jump (m)	42	1.90	0.23	1.59	1.60	1.66	1.70	1.71	1.77	1.92	2.00	2.07	2.10	2.10	2.18	2.26	1039	2.44	0.17	2.20	2.24	2.30	2.33	2.35	2.40	2.44	2.48	2.52	2.54	2.57	2.70	2.72
Sit-ups (repetitions)	41	51.15	10.12	32	37	41	45	47	52	53	55	56	60	61	61	65	1017	62.97	8.14	48	52	56	58	59	61	64	66	69	70	70	72	74
Pull-ups (repetitions)	18	4.06	2.24	1	1	2	3	3	3	3	5	6	6	6	7	-	977	15.22	5.85	7	8	10	11	12	13	14	16	19	20	21	24	25
Push-ups (repetitions)	15	25.73	7.12	7	12	20	20	25	28	30	30	30	30	30	31	-	-	-	-	-	-	-	-	-	-	-	-	-	-	-	-	-
Back and lumbar strength (kg)	12	245.68	93.73	100.0	106.3	140.8	163.3	187.3	202.2	253.3	284.2	329.1	342.1	347.9	364.4	-	573	274.29	66.13	166.0	191.2	215.1	228.0	237.0	256.0	276.0	295.6	300.0	300.0	314.4	358.8	393.5
Handgrip (kg)	40	77.86	10.10	63.1	65.2	70.0	71.0	72.1	74.5	76.1	78.1	79.8	81.5	89.6	93.9	97.8	949	108.68	17.52	80.0	86.0	94.0	96.0	99.0	103.0	108.0	112.5	118.0	121.0	124.0	132.0	139.0
Sit and reach (cm)	40	55.98	5.10	47	49	51	52	53	55	57	59	60	60	61	62	62	957	51.86	6.90	41	44	46	47	48	50	52	53	56	57	58	61	64
Cooper (m)	24	2153.58	326.22	1570	1690	1950	1980	1980	2010	2150	2200	2250	2300	2370	2743	2871	844	2832.80	253.22	2411	2500	2600	2650	2700	2750	2810	2900	3000	3010	3050	3150	3295
20 m shuttle run (laps)	16	62.75	17.89	34	40	43	47	56	60	60	63	67	78	85	92	-	179	89.23	16.37	63	70	77	80	80	82	90	91	97	102	102	110	115
Predicted *V*O_2_max (mL/kg/min)	40	41.08	8.37	25.3	32.3	33.1	33.9	36.3	37.9	40.9	42.1	47.6	47.6	47.6	53.6	56.5	1023	52.58	5.60	43.5	45.1	46.8	48.0	49.1	51.3	53.3	53.6	55.8	56.6	56.9	59.6	62.5
Total																																
60 m (s)	295	9.06	0.65	8.2	8.4	8.6	8.7	8.7	8.9	9.0	9.1	9.3	9.3	9.4	9.7	10.2	1411	7.73	0.40	7.1	7.2	7.4	7.5	7.5	7.6	7.7	7.8	7.9	8.0	8.1	8.2	8.4
30 m (s)	149	4.82	0.24	4.5	4.5	4.6	4.7	4.7	4.8	4.8	4.9	4.9	5.0	5.0	5.2	5.2	322	4.21	0.20	3.9	4.0	4.1	4.1	4.1	4.1	4.2	4.2	4.3	4.3	4.3	4.5	4.6
Slalom (s)	477	25.06	1.46	23.3	23.6	24.0	24.2	24.3	24.6	24.8	25.0	25.3	25.6	25.9	26.8	27.9	1894	22.85	1.23	21.1	21.5	21.8	22.0	22.2	22.4	22.7	23.0	23.3	23.5	23.8	24.4	25.0
Horizontal jump (m)	479	1.99	0.16	1.72	1.82	1.87	1.89	1.90	1.95	1.99	2.03	2.07	2.09	2.12	2.20	2.25	1908	2.43	0.16	2.20	2.24	2.30	2.33	2.35	2.39	2.43	2.47	2.50	2.53	2.56	2.65	2.72
Sit-ups (repetitions)	473	54.23	7.97	40	45	48	50	50	53	55	57	59	59	60	63	68	1890	61.44	8.49	46	50	54	56	57	60	62	65	67	69	70	72	73
Pull-ups (repetitions)	284	4.65	2.96	1	2	2	2	3	3	4	5	6	6	7	9	11	1848	15.34	5.99	7	8	10	11	12	13	14	16	19	20	21	25	25
Push-ups (repetitions)	149	27.04	6.50	12	15	23	24	26	30	30	30	30	30	30	32	34	-	-	-	-	-	-	-	-	-	-	-	-	-	-	-	-
Back and lumbar strength (kg)	148	226.36	71.91	104.0	120.5	156.8	177.3	190.8	210.2	230.0	248.4	266.5	276.5	293.5	328.0	345.21	880	283.27	67.16	177.0	199.0	224.0	235.4	247.0	268.0	287.5	300.0	303.4	316.2	333.6	373.3	398.1
Handgrip (kg)	448	70.79	11.55	54.0	58.0	61.6	63.3	64.6	67.6	70.0	72.0	75.6	76.9	78.5	85.0	92.0	1784	109.43	16.90	83.0	88.2	95.3	98.0	100.0	104.0	108.7	113.0	118.7	120.9	124.0	131.0	139.0
Sit and reach (cm)	462	54.24	6.52	44	47	50	50	51	53	54	56	57	58	59	62	65	1811	51.95	7.13	41	43	46	47	48	50	52	54	56	57	58	61	63
Cooper (m)	314	2349.78	254.40	1969	2100	2200	2200	2220	2300	2350	2400	2450	2500	2550	2653	2700	1540	2826.67	248.95	2400	2500	2600	2650	2700	2750	2805	2900	3000	3000	3050	3150	3250
20 m shuttle run (laps)	151	59.70	13.37	39	42	47	50	53	57	60	63	66	67	70	77	83	367	86.76	16.10	59	68	73	76	80	82	86	91	93	97	102	108	113
Predicted *V*O_2_max (mL/kg/min)	465	42.85	5.74	33.4	35.7	37.9	39.3	40.1	41.6	42.4	44.6	46.8	47.6	47.6	49.8	51.2	1907	52.36	5.47	43.5	44.8	47.3	48.0	49.1	51.3	52.4	53.6	55.8	56.2	56.9	59.6	61.37

Key: M, mean; SD, standard deviation; fit (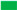) and unfit (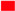) according to the cut-off values of prerequisite fitness tests for admission to the Portuguese Police Academy (i.e., the battery of physical fitness evaluations).

**Table 5:** Effect of the 4-year training program on physical fitness of police academy cadets by gender.

Variables	Delta (Δ)	Female							Male						
		Univariate Test	Mean Dif.	SEM	95% CI		Effect Size	*Sig.*	Univariate Test	Mean Dif.	SEM	95% CI		Effect Size	*Sig.*
					Lower	Higher						Lower	Higher		
Body mass (kg)	Δ (T1-T0)	*F*(4,146.78) = 1.503, *p* = 0.204	−0.347	0.568	−1.783	1.088	0.04	1.000	***F*(4,446.98) =** **16.796, *p* < 0.001**	0.332	0.345	−0.533	1.196	0.04	1.000
	Δ (T2-T0)		−0.760	0.580	−2.228	0.708	0.09	0.771		1.631	0.371	−0.702	2.561	0.20	**<0.001**
	Δ (T3-T0)		−1.370	0.755	−3.279	0.539	0.16	0.286		1.993	0.400	−0.989	2.996	0.24	**<0.001**
	Δ (T4-T0)		−1.367	0.708	−3.157	0.424	0.16	0.222		3.268	0.436	−2.174	4.363	0.40	**<0.001**
60 m (s)	Δ (T1-T0)	***F*(4,202.92) =** **12.262, *p* < 0.001**	−0.145	0.055	−0.283	−0.007	0.21	**0.034**	***F*(4,1012.44) =** **59.408, *p* < 0.001**	−0.144	0.019	−0.192	−0.095	0.33	**<0.001**
	Δ (T2-T0)		−0.233	0.060	−0.385	−0.082	0.33	**0.001**		−0.208	0.021	−0.262	−0.155	0.48	**<0.001**
	Δ (T3-T0)		−0.385	0.061	−0.539	−0.232	0.55	**<0.001**		−0.305	0.021	−0.358	−0.252	0.71	**<0.001**
	Δ (T4-T0)		−0.324	0.063	−0.484	−0.164	0.46	**<0.001**		−0.226	0.021	−0.278	−0.173	0.53	**<0.001**
30 m (s)	Δ (T1-T0)	*F*(4,96.87) = 0.923, *p* = 0.454	−0.004	0.028	−0.075	0.067	0.01	1.000	***F*(4,229.49) =** **7.293, *p* < 0.001**	−0.113	0.024	−0.174	−0.052	0.45	**<0.001**
	Δ (T2-T0)		−0.027	0.029	−0.100	0.047	0.10	1.000		−0.098	0.026	−0.163	−0.032	0.39	**0.001**
	Δ (T3-T0)		0.015	0.034	−0.070	0.101	0.06	1.000		−0.097	0.026	−0.163	−0.030	0.39	**0.001**
	Δ (T4-T0)		−0.070	0.066	−0.237	0.097	0.26	1.000		−0.151	0.037	−0.243	−0.059	0.60	**<0.001**
Slalom (s)	Δ (T1-T0)	***F*(4,345.17) =** **17.761, *p* < 0.001**	−0.507	0.099	−0.756	−0.259	0.30	**<0.001**	***F*(4,1368.74) =** **118.331, *p* < 0.001**	−0.611	0.047	−0.728	−0.493	0.43	**<0.001**
	Δ (T2-T0)		−0.694	0.103	−0.953	−0.436	0.41	**<0.001**		−0.832	0.048	−0.951	−0.712	0.59	**<0.001**
	Δ (T3-T0)		−0.758	0.112	−1.040	−0.476	0.45	**<0.001**		−0.856	0.051	−0.983	−0.730	0.60	**<0.001**
	Δ (T4-T0)		−0.777	0.125	−1.091	−0.463	0.46	**<0.001**		−0.905	0.054	−1.041	−0.769	0.64	**<0.001**
Horizontal jump (m)	Δ (T1-T0)	***F*(4,352.13) =** **3.631, *p* = 0.006**	0.029	0.013	−0.003	0.061	0.18	0.090	***F*(4,1386.35) =** **44.504, *p* < 0.001**	0.051	0.007	0.034	0.067	0.30	**<0.001**
	Δ (T2-T0)		0.023	0.013	−0.011	0.057	0.14	0.349		0.061	0.007	0.044	0.078	0.36	**<0.001**
	Δ (T3-T0)		0.052	0.015	0.015	0.088	0.33	**0.002**		0.083	0.007	0.065	0.101	0.49	**<0.001**
	Δ (T4-T0)		0.040	0.016	0.000	0.081	0.25	0.052		0.076	0.008	0.057	0.095	0.45	**<0.001**
Sit-ups 60 s (repetitions)	Δ (T1-T0)	***F*(4,350.19) =** **9.600, *p* < 0.001**	0.341	0.668	−1.337	2.019	0.01	1.000	***F*(4,1412.13) =** **66.801, *p* < 0.001**	2.241	0.366	1.326	3.156	0.26	**<0.001**
	Δ (T2-T0)		3.263	0.685	1.547	4.984	0.38	**<0.001**		5.013	0.374	4.077	5.949	0.58	**<0.001**
	Δ (T3-T0)		3.160	0.741	1.299	5.022	0.37	**<0.001**		4.840	0.391	3.862	5.819	0.56	**<0.001**
	Δ (T4-T0)		2.596	0.833	0.506	4.687	0.30	**0.008**		4.934	0.405	3.921	5.946	0.57	**<0.001**
Pull-ups (repetitions)	Δ (T1-T0)	*F*(4,203.07) = 1.830, *p* = 0.124	0.710	0.291	−0.024	1.445	0.28	0.063	***F*(4,1333.89) =** **33.610, *p* < 0.001**	1.248	0.244	0.638	1.858	0.22	**<0.001**
	Δ (T2-T0)		0.254	0324	−0.562	1.070	0.10	1.000		1.830	0.260	1.179	2.480	0.32	**<0.001**
	Δ (T3-T0)		0.399	0.358	−0.503	1.301	0.15	1.000		2.789	0.267	2.122	3.456	0.49	**<0.001**
	Δ (T4-T0)		0.041	0.356	−0.857	0.939	0.02	1.000		2.456	0.285	1.744	3.169	0.43	**<0.001**
Push-ups (repetitions)	Δ (T1-T0)	*F*(4,104.78) = 1.678, *p* = 0.161	1.069	1.045	−1.606	3.743	0.14	1.000	-	-	-	-	-	-	-
	Δ (T2-T0)		−1.718	1.077	−4.468	1.032	0.23	0.458		-	-	-	-	-	-
	Δ (T3-T0)		−0.487	1.208	−3.557	2.583	0.06	1.000		-	-	-	-	-	-
	Δ (T4-T0)		−0.314	1.992	−5.357	4.730	0.04	1.000		-	-	-	-	-	-
Back lumbar strength (kg)	Δ (T1-T0)	***F*(4,91.11) =** **12.033, *p* < 0.001**	24.059	11.156	−4.371	52.490	0.37	0.135	***F*(4,605.09) =** **79.376, *p* < 0.001**	38.013	5.007	25.470	50.556	0.63	**<0.001**
	Δ (T2-T0)		53.598	12.144	22.719	84.477	0.83	**<0.001**		50.962	4.882	38.735	63.189	0.84	**<0.001**
	Δ (T3-T0)		73.037	13.924	37.593	108.482	1.13	**<0.001**		67.208	5.674	52.994	81.422	1.11	**<0.001**
	Δ (T4-T0)		89.814	15.160	51.224	128.404	1.38	**<0.001**		92.102	5.504	78.314	105.890	1.51	**<0.001**
Handgrip (kg)	Δ (T1-T0)	*F*(4,333.85) = 1.433, *p* = 0.223	1.877	1.081	−0.839	4.593	0.17	0.334	***F*(4,1299.43) =** **31.521, *p* < 0.001**	3.870	0.749	1.997	5.743	0.22	**<0.001**
	Δ (T2-T0)		2.343	1.146	−0.535	5.221	0.21	0.167		6.671	0.779	4.722	8.620	0.38	**<0.001**
	Δ (T3-T0)		1.882	1.220	−1.180	4.945	0.17	0.494		6.130	0.810	4.103	8.156	0.35	**<0.001**
	Δ (T4-T0)		2.555	1.357	−0.852	5.962	0.23	0.242		8.590	0.843	6.481	10.700	0.49	**<0.001**
Sit and reach (m)	Δ (T1-T0)	*F*(4,337.06) = 1.537, *p* = 0.191	0.224	0.370	−0.707	1.154	0.03	1.000	***F*(4,1317.43) =** **3.792, *p* = 0.005**	−0.480	0.265	−1.144	0.183	0.07	0.282
	Δ (T2-T0)		0.752	0.375	−0.188	1.693	0.11	0.181		0.289	0.264	−0.372	0.950	0.04	1.000
	Δ (T3-T0)		0.695	0.695	−0.332	1.722	0.10	0.360		−0.631	0.275	−1.318	0.056	0.09	0.087
	Δ (T4-T0)		0.762	0.762	−0.394	1.918	0.11	0.395		−0.379	0.286	−1.096	0.337	0.05	0.742
Cooper (m)	Δ (T1-T0)	*F*(4,229.30) = 2.003, *p* = 0.095	−19.351	26.944	−87.179	48.477	0.07	1.000	***F*(4,1122.15) =** **17.080, *p* < 0.001**	7.569	12.162	−22.857	37.994	0.03	1.000
	Δ (T2-T0)		−17.378	27.974	−87.813	53.057	0.07	1.000		31.821	12.264	1.140	62.502	0.14	**0.038**
	Δ (T3-T0)		−1.491	30.636	−78.627	73.645	0.01	1.000		−24.476	13.166	−57.414	8.462	0.11	0.253
	Δ (T4-T0)		−82.044	32.012	−162.638	−1.450	0.31	**0.044**		−76.786	13.363	−110.218	−43.354	0.34	**<0.001**
20 m shuttle run (laps)	Δ (T1-T0)	*F*(4,111.39) = 1.056, *p* = 0.382	3.387	2.058	−1.866	8.640	0.25	0.414	*F*(4,259.99) = 0.658, *p* = 0.622	−1.263	1.714	−5.586	3.059	0.08	1.000
	Δ (T2-T0)		3.002	2.132	−2.432	8.435	0.22	0.650		−0.991	1.853	−5.654	3.673	0.07	1.000
	Δ (T3-T0)		1.031	2.454	−5.199	7.261	0.08	1.000		−2.963	1.941	−7.842	1.917	0.20	0.512
	Δ (T4-T0)		4.588	4.012	−5.567	14.744	0.34	1.000		−2.561	2.524	−8.896	3.775	0.17	1.000
Predicted *V*O_2max_ (mL/kg/min)	Δ (T1-T0)	*F*(4,340.93) = 1.334, *p* = 0.257	−0.213	0.508	−1.489	1.062	0.04	1.000	***F*(4,1413.50) =** **12.037, *p* < 0.001**	−0.116	0.249	−0.739	0.508	0.02	1.000
	Δ (T2-T0)		0.380	0.529	−0.947	1.707	0.07	1.000		0.643	0.255	−0.006	1.281	0.13	**0.047**
	Δ (T3-T0)		0.970	0.573	−0.469	2.409	0.17	0.366		−0.272	0.268	−0.942	0.398	0.28	1.000
	Δ (T4-T0)		0.041	0.636	−1.555	1.637	0.01	1.000		−1.306	0.278	−2.000	−0.612	0.26	**<0.001**

Key: CI, confidence interval; SEM, standard error of the mean; Effect size thresholds (used to assess the magnitude of the difference in means) were interpreted as (i) small, ≤0.19; (ii) medium, 0.20–0.49; (iii) high, 0.50–0.79; and (iv) very high, ≥0.80 [36].

## Data Availability

The data presented in this study are available upon reasonable request from the corresponding author. The data are not publicly available due to privacy and ethical restrictions.
